# Efficacy of Hydrocortisone, Povidone-Iodine, and Normal Saline as an Irrigating Solution During Surgical Removal of Impacted Mandibular Third Molars: A Randomized Controlled Trial

**DOI:** 10.7759/cureus.53370

**Published:** 2024-02-01

**Authors:** Faheem Ahmed, Shridhar D Baliga, Sulakshana S Baliga, Pranjal Rathi, Gaurav Jha

**Affiliations:** 1 Oral and Maxillofacial Surgery, KAHER's (KLE Academy of Higher Education and Research's) KLE Vishwanath Katti Institute of Dental Sciences, Belagavi, IND; 2 Community Medicine, KAHER'S (KLE Academy of Higher Education and Research's) Jawaharlal Nehru Medical College, Belagavi, IND; 3 Department of Oral and Maxillofacial Surgery, Bharati Vidyapeeth Deemed to be University Dental College and Hospital, Pune, IND

**Keywords:** irrigation, postoperative, mandibular third molar(s), surgical extraction, trismus, pain, edema, povidone iodine, normal saline, hydrocortisone

## Abstract

Background

The surgical removal of mandibular third molars is one of the most common procedures in dentistry. Researchers have extensively studied the treatment of postoperative sequelae such as pain, edema, trismus, and alveolar osteitis throughout the past six decades. Many approaches have been used to address clinical difficulties after third molar surgery, including various flap designs and irrigating solutions. The aim of this study was to compare the effects of three irrigating solutions, hydrocortisone, povidone-iodine, and normal saline, on pain, trismus, and edema following surgical removal of the impacted mandibular third molar.

Methodology

The study involved 105 participants who required surgical extraction of mandibular third molars. The patients' ages ranged from 18 to 40 years, and they fulfilled the inclusion criteria. Using a simple random sampling technique, they were divided into three groups (group 1: hydrocortisone, group 2: povidone-iodine, group 3: normal saline). The parameters evaluated were edema, pain, and trismus on the second and seventh postoperative days. All data were input into Microsoft Excel (Microsoft^®^ Corp., Redmond, USA) worksheets and analyzed using Stata 17.0 (StataCorp LLC, College Station, USA). The visual analog scale (VAS) score was used to measure postoperative pain, and postoperative swelling was measured using linear measurements from four fixed anatomical points and compared to preoperative values. To assess trismus, the inter-incisal distance was measured in millimeters with a caliper. A p-value of <0.01 was considered statistically significant.

Results

The mean VAS score for pain in group 1 was lower than the other two groups. The effect of group 1 was significant on the second postoperative day but insignificant on the seventh postoperative day for swelling. The effect of all three groups on trismus was significant on the second and seventh days.

Conclusions

Hydrocortisone as an irrigating solution showed promising results in managing postoperative swelling in the first 48 hours, but its effect gradually declined by the seventh postoperative day. Additionally, it was effective in controlling postoperative pain and trismus. This suggests that utilizing hydrocortisone as an irrigating solution, compared to povidone-iodine, has been proven to be a significantly effective option in reducing postoperative pain, edema, and trismus resulting from the surgical removal of impacted teeth.

## Introduction

Third molar eruption occurs most commonly between the ages of 17 and 26 [1]. Third molars are either partially erupted or fail to erupt completely, with a prevalence rate of 22.63 % globally [2]. The symptoms of the initial postoperative tissue reactions include pain, edema, trismus, and dysphagia, which have a major impact on the patient's quality of life [3,4]. Older people have a much higher incidence of experiencing prolonged postoperative morbidity than younger ones [4,5]. Irrigating solutions are widely used during surgical extractions to minimize bone damage, irrigate the surgical site, and improve the dentist's visual perception of the operating area.

Spies et al. were the first to employ hydrocortisone for the management of oral disorders related to local causes and oral symptoms of inflammatory systemic diseases [6].The most typical method of administering corticosteroids in oral surgery is oral administration, but it is contraindicated in patients with gastrointestinal problems. When the procedure is carried out under general anesthesia, hydrocortisone delivered through the intravenous route produces positive outcomes in postoperative edema and pain control [7]. Povidone-iodine (PVP-I) acts as a coolant during the extraction of an impacted lower third molar. Additionally, it has a hemostatic effect and leads to a decrease in postoperative edema [8]. Normal saline, an isotonic solution, is utilized for irrigation during impacted third molar treatments.

The purpose of this study was to evaluate and compare the efficacy of using hydrocortisone, povidone-iodine, and normal saline as an irrigating solution in terms of postoperative edema, postoperative pain, and the level of trismus during surgical removal of the impacted mandibular third molar.

## Materials and methods

The study involved 105 participants who required surgical extraction of the mandibular third molar. Written informed consent was obtained from all participants. Ethical approval was obtained from the ethical committee of KLE Vishwanath Katti Institute of Dental Sciences, Belagavi, with reference number 1465 dated 5/5/2021, and the study was registered with the Clinical Trial Registry of India (CTRI) with reference number REF/2023/09/073554 prior to the study.

Study design and sample size estimation

This is a randomized controlled trial. The sample size calculation was based on parameters obtained from previous studies using the formula with a 90% confidence interval Z1-α/2 = 1.64, at 80% power Z1-β = 0.85, the standard deviation in the first group (hydrocortisone) was 3.16, the standard deviation in the second group (povidone-iodine) was 3.46. The mean value of the first group was 5.33, while the mean value of the second group was 3.33. Therefore, n in each group was 35. The study comprised 105 patients with mandibular impacted third molars, who were randomly selected using the envelope method, and it was single-blinded for allocation concealment. All the patients were diagnosed by established clinical and radiographic parameters and were alternatively grouped into three groups irrespective of age, sex, difficulty in impaction, and their response to various drugs to eliminate bias.

Study participants were categorized into three groups - group 1: third molar surgeries with hydrocortisone irrigation (n=35), group 2: third molar surgeries performed using povidone-iodine irrigation (n=35), group 3: third molar surgeries done with normal saline irrigation (control group) (n=35)

Criteria for patient selection

**Figure 1:**
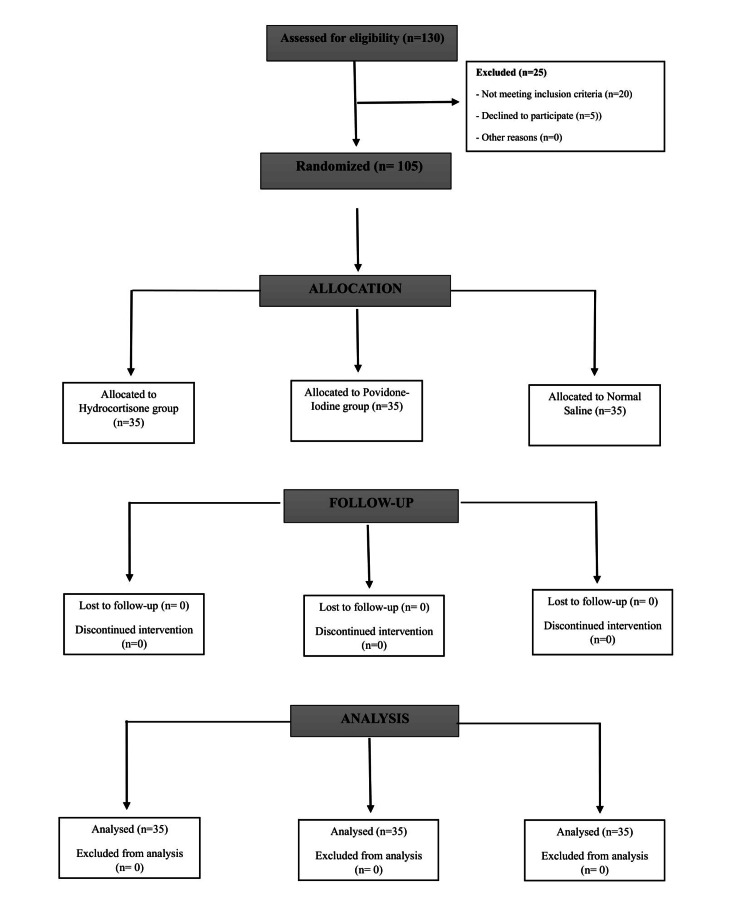
Consolidated Standards of Reporting Trials (CONSORT) flow diagram for randomized controlled trial of hydrocortisone, povidone-iodine and normal saline as an irrigating solution during surgical removal of impacted mandibular third molar

Patients aged 18-40 years who had not used any antibiotic/antimicrobial or anti-inflammatory drugs in the week prior to the surgery and patients without a history of smoking were included. Additionally, patients with teeth scoring five to six on Pederson's difficulty index were included in the study. The following patients were excluded from the study: those who were unwilling to participate, those with systemic disorders (such as diabetes, hypertension, cardiovascular diseases, chronic liver disease, chronic kidney disease, bleeding disorders, and immunocompromised patients), those with a history of radiation therapy, and pregnant or lactating female subjects. This study followed Consolidated Standards of Reporting Trials (CONSORT) guidelines with the flow diagram shown in Figure 1.

Method of operation

Patients with mandibular impacted third molars diagnosed by established clinical and radiographic parameters and who met the inclusion criteria were divided into three groups of 35 each, which were randomly selected by envelope method. The surgical procedure on the assigned patients was performed in the oral surgery unit by the same experienced surgeon. The preoperative inter-incisal distance and facial measurements were noted in millimeters. Local anesthesia was given by blocking the inferior alveolar nerve, lingual nerve, and long buccal nerve with 2% lignocaine plus adrenaline 1:80,000. A full-thickness incision was made to prepare a muco-periosteal flap. The flap was elevated and reflected, and bone guttering (tooth sectioning if required) was performed using a bur on a straight handpiece under abundant irrigation with hydrocortisone 500 mg concentration in 250 ml normal saline for group 1, povidone-iodine 0.5 mg concentration per ml in 250 ml of normal saline for group 2 and 0.9% concentration of 250 ml of normal saline for group 3 [8,9]. After completing the extraction, curettage of the socket was performed to remove any unhealthy granulation tissue. The extraction socket was inspected for any sharp bony margins and removed if present, followed by copious irrigation. The flap was repositioned and sutured with 3-0 silk sutures. A pressure pack was placed on the extraction site. All patients received post-extraction instructions. Patients of all three groups were prescribed the following drugs: antibiotic (amoxicillin capsules of 500 mg) thrice daily for five days, analgesic (paracetamol tablets of 650 mg) twice daily for three days, antacid (pantoprazole tablets of 20 mg) once a day for five days, ibuprofen tablets of 400mg was given as a rescue drug.

Evaluation criteria

**Figure 2:**
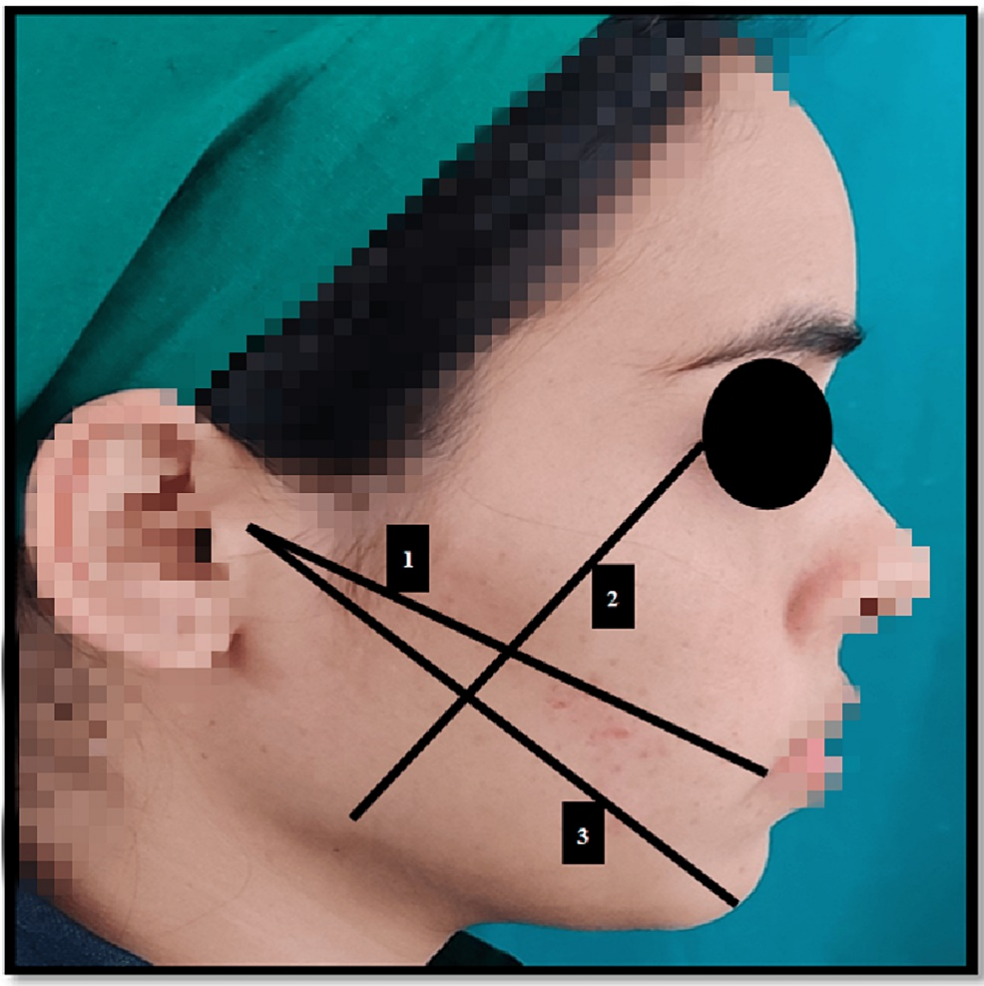
Edema measurements 1 - from the tragus to the corner of the mouth; 2 - from the lateral canthus of the eye to the angle of the mandible; 3 - from the tragus to the soft tissue pogonion

All the patients were provided with a visual analog scale (VAS) data sheet with a score of 0 to 10 (0 - no pain to 10 - very severe pain) for pain. VAS scores and the number of tablets taken were collected at two time points: the second and seventh postoperative days. Postoperative edema was evaluated using linear measurements compared to preoperative values. To evaluate the swelling, three linear distances in millimeters for four fixed anatomical points were measured, as depicted in Figure 2. The linear distances are as follows: 1 - distance from the tragus to the corner of the mouth; 2 - distance from the lateral canthus of the eye to the angle of the mandible; 3 - distance from the tragus to the soft tissue pogonion. These measurements were obtained using a ribbon ruler on the side of the surgery [10]. The measurements were taken at three time points: prior to the surgery and on the second and seventh postoperative days. To evaluate trismus, the mouth opening was checked by measuring the inter-incisal distance in millimeters with a caliper. The pain, swelling, and trismus were measured preoperatively on the second and the seventh preoperative days. 

Statistical analysis

All data were analyzed using Stata 17.0 (StataCorp LLC, College Station, USA). Descriptive statistical analysis was used for demographic details. Comparisons between the three groups at different periods were conducted using the Kruskal-Wallis test, as the data were not normally distributed. The comparison between two time periods for the three groups was conducted using the Wilcoxon signed-rank test, while for three time periods, it was conducted using repeated measures ANOVA.

## Results

The average age of the patients across the three groups was 26.93 years, and the comparison was done using the Kruskal-Wallis test. The comparison between age groups was not statistically significant (p=0.494), which denotes no baseline difference between the three groups. There were a total of 49 males and 56 females in the three groups, and no statistically significant difference was found between them at the baseline. The assessment of pain was done using VAS scores for the hydrocortisone, povidone-iodine, and control groups, which were calculated on day 2 and day 7. The results were analyzed using the Kruskal-Wallis test and are depicted in Table 1. The difference between the three groups was found to be statistically significant (p<0.001) for both day 2 and day 7. Moreover, the comparison of the mean difference between day 2 and day 7 for hydrocortisone, povidone-iodine, and normal saline was also found to be statistically significant.

**Table 1:** Comparison of hydrocortisone, povidone-iodine and control (normal saline) groups with VAS scores at the second and sevenths postoperative days using Kruskal-Wallis test *p<0.05 statistically significant Visual analog scale (VAS) scores: 0=none, 1-3=mild, 4-6=moderate, 7-10=severe

Time points	Groups	Mean	SD	H-value	p-value
Day 2	Hydrocortisone	4.82	0.70	34.980	<0.001*
	Povidone-iodine	4.57	0.60		
	Normal saline	5.71	0.45		
Day 7	Hydrocortisone	2.62	0.59	13.091	<0.001*
	Povidone-iodine	2.82	0.70		
	Normal saline	3.34	0.48		
Day 2 - day 7	Hydrocortisone	2.20	0.11	21.889	<0.001*
	Povidone-iodine	1.75	-0.10		
	Normal saline	2.37	-0.03		

When the assessment of the mean VAS score for pain was conducted on postoperative day 2 and day 7, and a comparison was made for the three groups using the Wilcoxon signed-rank test, as mentioned in Table 2, it was found that the mean VAS score was lower for the hydrocortisone group on day 7 when compared to the others. The difference between the two time points was found to be statistically significant for all of them, with a consistent p-value of less than 0.05 (p<0.001).

**Table 2:** Comparison of VAS scores at the second and seventh postoperative days in hydrocortisone, povidone-iodine and control (normal saline) groups using Wilcoxon signed-rank test *p<0.05 statistically significant Visual analog scale (VAS) scores: 0=none, 1-3=mild, 4-6=moderate, 7-10=severe

Groups	Time points	Mean	SD	Z-value	p-value
Hydrocortisone	Day 2	4.82	0.70	5.406	<0.001*
	Day 7	2.62	0.59		
Povidone-iodine	Day 2	4.57	0.60	5.387	<0.001*
	Day 7	2.82	0.70		
Normal saline	Day 2	5.71	0.45	5.282	<0.001*
	Day 7	3.34	0.48		

When the assessment for swelling was done preoperatively and postoperatively on day 2 and day 7, it was found that the comparison of swelling scores between the three groups (hydrocortisone, povidone-iodine, and normal saline) was done at three different time points: preoperative stage, day 2, and day 7, using the Kruskal-Wallis test. The results are provided in Table 3. The comparative results between the three groups were not found to be statistically significant at the preoperative stage and day 7. However, the comparison between them on day 2 was statistically significant. Even for the comparison of the mean differences between the three groups at preoperative stage to day 7 and day 2 to day 7, it was found to be statistically insignificant. However, for the preoperative stage to day 2, it was found to be statistically significant.

**Table 3:** Comparison of hydrocortisone, povidone-iodine and control (normal saline) groups with swelling scores at the preoperative stage, postoperative second and seventh days using Kruskal-Wallis test *p<0.05 statistically significant

Variable	Groups	Mean (mm)	SD (mm)	SE (mm)	H-value	P-value
Preoperative stage	Hydrocortisone	126.27	6.56	1.109	1.802	0.170
	Povidone-iodine	124.26	6.33	1.071		
	Normal saline	123.04	8.45	1.429		
Day 2	Hydrocortisone	128.90	5.80	0.982	2.278	0.010*
	Povidone-iodine	129.28	6.58	1.113		
	Normal saline	132.18	8.40	1.420		
Day 7	Hydrocortisone	125.99	6.57	1.111	0.051	0.941
	Povidone-iodine	125.53	5.79	0.979		
	Normal saline	126.08	8.66	1.463		
Preoperative stage - day 2	Hydrocortisone	-2.63	0.76	0.127	0.893	0.022*
	Povidone-iodine	-5.02	-0.25	-0.042		
	Normal saline	-9.14	0.05	0.009		
Preoperative stage - day 7	Hydrocortisone	0.28	-0.01	-0.002	1.366	0.785
	Povidone-iodine	-1.27	0.54	0.092		
	Normal saline	-3.04	-0.21	-0.034		
Day 2 - day 7	Hydrocortisone	2.91	-0.77	-0.129	1.795	0.658
	Povidone-iodine	3.75	0.79	0.134		
	Normal saline	6.1	-0.26	-0.043		

During the comparison of swelling for the three groups, measurements were taken at three different time points: preoperative stage, day 2, and day 7. The statistical significance between them was analyzed using the repeated measures ANOVA test, and the results are presented in Table 4. Additionally, the comparison between two individual time points for each group was conducted using the Wilcoxon signed-rank test. For the hydrocortisone group, the comparison between the preoperative stage and day 2, and between day 2 and day 7, was found to be statistically significant. For the povidone-iodine and normal saline groups, the comparison between all three combinations (i.e., preoperative stage to day 2, preoperative stage to day 7, and day 2 to day 7) was found to be statistically significant. Overall, the differences between the three time points for all three groups were found to be statistically significant based on the repeated measures ANOVA.

**Table 4:** Comparison of the preoperative stage, the second and seventh postoperative days with swelling scores in hydrocortisone, povidone-iodine and control (normal saline) groups using repeated measures ANOVA test *p<0.05 statistically significant ^1^ statistical significance for the Wilcoxon signed-rank test; ^2^ statistical significance for repeated measures ANOVA test

Groups	Time points	Mean (mm)	SD (mm)	Mean Diff. (mm)	SD Diff. (mm)	p-value^1^	Repeated measures	
							F-value	p-value^2^
Hydrocortisone	Preoperative stage	126.27	6.56	2.633	0.580	<0.001*	59.318	<0.001*
	Day 2	128.90	5.80					
	Preoperative stage	126.27	6.56	0.279	0.186	0.429		
	Day 7	125.99	6.57					
	Day 2	128.90	5.80	2.911	0.770	<0.001*		
	Day 7	125.99	6.57					
Povidone-iodine	Preoperative stage	124.26	6.33	5.019	0.250	<0.001*	230.777	<0.001*
	Day 2	129.28	6.58					
	Preoperative stage	124.26	6.33	1.268	0.454	0.025*		
	Day 7	125.53	5.79					
	Day 2	129.28	6.58	3.946	0.790	<0.001*		
	Day 7	125.53	5.79					
Normal saline	Preoperative stage	123.04	8.45	9.133	0.213	<0.001*	926.79	<0.001*
	Day 2	132.18	8.40					
	Preoperative stage	123.04	8.45	3.038	0.264	<0.001*		
	Day 7	126.08	8.66					
	Day 2	132.18	8.40	6.095	0.256	<0.001*		
	Day 7	126.08	8.66					

Table 5 describes the results of the comparison of inter-incisal distance for trismus between hydrocortisone, povidone-iodine, and normal saline at the preoperative stage, day 2, and day 7 using the Kruskal-Wallis test. The comparison between all three groups at all three time points was found to be statistically significant (p<0.001). The mean difference between the time points of the three groups (preoperative stage to day 2, preoperative stage to day 7, and day 2 to day 7) was also calculated, and their comparison yielded a statistically significant difference as well.

**Table 5:** Comparison of hydrocortisone, povidone-iodine and control (normal saline) groups with inter-incisal distance at the preoperative stage, the second and seventh postoperative days using Kruskal-Wallis test *p<0.05 statistically significant

Variable	Groups	Mean (mm)	SD (mm)	SE (mm)	H-value	p-value
Preoperative stage	Hydrocortisone	42.20	7.58	1.281	12.044	<0.001*
	Povidone-iodine	40.60	3.11	0.526		
	Normal saline	36.54	2.62	0.444		
Day 2	Hydrocortisone	33.14	5.99	1.013	48.947	<0.001*
	Povidone-iodine	34.37	3.40	0.575		
	Normal saline	24.94	2.96	0.502		
Day 7	Hydrocortisone	40.62	7.35	1.243	30.449	<0.001*
	Povidone-iodine	38.65	3.32	0.562		
	Normal saline	31.88	2.70	0.457		
Preoperative stage - day 2	Hydrocortisone	9.06	1.59	0.268	16.346	<0.001*
	Povidone-iodine	6.23	-0.29	-0.049		
	Normal saline	11.6	-0.34	-0.058		
Preoperative stage - day 7	Hydrocortisone	1.58	0.23	0.038	8.904	<0.001*
	Povidone-iodine	1.95	-0.21	-0.036		
	Normal saline	4.66	-0.08	-0.013		
Day 2 - day 7	Hydrocortisone	-7.48	-1.36	-0.23	-3.228	<0.001*
	Povidone-iodine	-4.28	0.08	0.013		
	Normal saline	-6.94	0.26	0.045		

The overall comparison of the three groups at different time points for inter-incisal opening was conducted using the repeated measures ANOVA test and is denoted in Table 6. The comparison of the differences between the different combinations of two time points, i.e., preoperative stage to day 2, preoperative stage to day 7, and day 2 to day 7 for all three groups, was found to be statistically significant (p<0.001). Similarly, the overall comparison of the three time points for all three groups was found to be statistically significant (p<0.001).

**Table 6:** Comparison of the preoperative stage, the second and seventh postoperative days with inter-incisal distance in hydrocortisone, povidone-iodine and control (normal saline) groups using repeated measures ANOVA test *p<0.05  statistically significant ^1^ statistical significance for the Wilcoxon signed-rank test; ^2 ^statistical significance for repeated measures ANOVA test

Groups	Time points	Mean (mm)	SD (mm)	Mean Diff. (mm)	SD Diff. (mm)	p-value^1^	Repeated measures	
							F-value	p-value^2^
Hydrocortisone	Preoperative stage	42.20	7.58	9.057	0.755	<0.001*	73.629	<0.001*
	Day 2	33.14	5.99					
	Preoperative stage	42.20	7.58	1.571	0.324	<0.001*		
	Day 7	40.62	7.35					
	Day 2	33.14	5.99	7.486	0.621	<0.001*		
	Day 7	40.62	7.35					
Povidone-iodine	Preoperative stage	40.60	3.11	6.229	0.164	<0.001*	703.061	<0.001*
	Day 2	34.37	3.40					
	Preoperative stage	40.60	3.11	1.943	0.142	<0.001*		
	Day 7	38.65	3.32					
	Day 2	34.37	3.40	4.286	0.162	<0.001*		
	Day 7	38.65	3.32					
Normal saline	Preoperative stage	36.54	2.62	11.60	0.446	<0.001*	331.072	<0.001*
	Day 2	24.94	2.96					
	Preoperative stage	36.54	2.62	4.657	0.323	<0.001*		
	Day 7	31.88	2.70					
	Day 2	24.94	2.96	6.943	0.406	<0.001*		
	Day 7	31.88	2.70					

## Discussion

The removal of the mandibular third molar is one of the most common procedures in oral and maxillofacial surgery. Patients usually complain of postoperative swelling, pain, and trismus associated with the inflammatory response to surgical trauma as the main factors affecting their quality of life in the immediate postoperative period [11]. The fundamental objective of employing irrigating solutions during the surgical extraction of impacted third molars is to avoid heat-induced permanent bone necrosis. Normal saline, which is commonly used as an intraoperative irrigating solution, has a cleaning effect that aids in the healing of wounds but does not immediately contribute to postoperative healing [12].

To reduce postoperative discomfort, different irrigating solutions and pharmacological management have been frequently discussed in the literature. In oral and maxillofacial surgery, povidone-iodine has been used mainly as an irrigant for alveolar sockets after extraction [13]. In a 2011 study, Arakeri and Brennan utilized 0.5 mg/ml PVP-I as the cooling and irrigating solution for the surgical extraction of impacted third molars. They observed that it significantly reduced postoperative edema [8]. In this context, they hypothesized that the impact is brought on by chemotaxis and leukotriene B4 suppression, which prevent neutrophil accumulation. An additional benefit of PVP-I irrigation is the enhancement of the "anionic chemo-mechanical effects" of saline solution, which accelerates bone/tooth cutting rate by adding an additional anion (iodine) to saline [14].

Corticosteroids in oral surgery have been studied since 1952 [6] when hydrocortisone was used to reduce postoperative discomfort. Since that time, a number of additional corticosteroids, dosages, and administration methods have been researched to offer a more comfortable postoperative period and enhance the quality of life for patients. It is uncertain how hydrocortisone works to reduce inflammation. It is presumably connected to both cellular effects and how they affect the microvasculature [15].

The body naturally produces hydrocortisone (cortisol), with a typical daily production in an adult of between 15 and 30 mg, but it can be produced in amounts up to 300 mg in times of need [16,17]. Corticosteroids need to be administered at higher dosages than basal secretion in order to have an anti-inflammatory effect. However, there hasn't been a single study that can demonstrate the ideal corticosteroid dosage, ideal method of administration, or optimal timing. The most popular method of drug administration in oral surgery is oral administration. Additionally, the intravenous approach is employed, particularly when the third molar extraction is carried out under general anesthesia, because of its effectiveness in reducing pain and edema, as well as in preventing nausea and vomiting. This might be regarded as one of the finest methods of administration [9,18,19].

However, as these procedures are typically done under local anesthesia in an outpatient setting, the oral route continues to be the most popular choice. Additionally, it guarantees quick and nearly total absorption. Although the relative merits of different routes are always in discordance, corticosteroids should be used if considerable postoperative soft tissue edema is anticipated [9,18]. In this context, the current study was conducted to assess the effectiveness of hydrocortisone, povidone-iodine, and normal saline as irrigating solutions during impacted mandibular third molar surgery. Inconsistent findings from numerous studies have been discovered regarding the usage of corticosteroids to alleviate postoperative discomfort. Fortunately, some trials using a single dose of prednisolone or dexamethasone postoperatively showed substantial differences in edema, trismus, and pain [18,20], while others found conflicting results [21,22]. On the contrary, some studies have shown that using hydrocortisone as an intraoperative irrigating solution has a rapid and effective effect on managing postoperative edema [9,23].

In the present study, it was found that hydrocortisone, as an intraoperative irrigating solution, was more effective than povidone-iodine and normal saline in controlling postoperative swelling on the second day after surgical removal of impacted mandibular third molar, with a statistically significant difference. However, the comparison of the same irrigating solutions on postoperative day 7 was found to be statistically insignificant. Furthermore, trismus develops as a result of muscular spasms caused by inflammation. In addition, the edema-induced inflammatory process compresses nerve tissues, limiting movement and creating an unpleasant sensation that can vary from discomfort to severe pain [24]. Corticosteroids' capacity to diminish edema could enhance its analgesic impact by reducing pain and trismus brought on by tissue tension. This idea is compatible with Rizqiawan et al.'s claim that postoperative edema, trismus, and pain are associated and last longer in older patients compared to younger ones since inflammatory responses slow with age. Controlling the inflammatory process and edema would greatly relieve pain and trismus [25].

One of the most commonly used tools for measuring pain intensity is the VAS score, which has been proven to be a reliable and consistent way to measure unique pain. It is a straightforward, subdued, reproducible, and widely accepted method to evaluate pain [26]. It has to be noted that, in the studies reviewed, pain was assessed subjectively and was not the only primary outcome measured. This led to a variety of pain outcomes following third molar operations. The degree of pain is also influenced by a number of variables, including surgical trauma, a person's pain threshold, and their psychological well-being [20]. The current study showed a statistically significant result in postoperative pain control in both the study groups when compared to normal saline. According to the VAS score, there was a meaningful reduction in pain seen in study group 2 (PVP-I) on the second postoperative day, followed by the hydrocortisone group. Conversely, study group 1 (hydrocortisone) manifested a better response on the seventh postoperative day in comparison to the rest.

In third molar extractions, several authors have demonstrated that intraoperative irrigations with the povidone-iodine solution also reduced trismus. They contend that a povidone-iodine solution, when used in relatively low concentration, exhibited anti-inflammatory properties in addition to its antibacterial properties [8,13,26]. In a study, Tiigimae-Saar et al. discovered that the combination of a single dosage of prednisolone and etoricoxib is effective at treating trismus, edema, and postoperative pain following third molar surgery [18]. In the current analysis, when trismus was examined between the study and control group, both study groups showed a statistically significant decrease in trismus. Study group 2 (povidone-iodine) patients had the maximum inter-incisal distance on the second postoperative day, which was followed by study group 1 (hydrocortisone). However, hydrocortisone participants had the least overall trismus on the seventh postoperative day compared to the rest. So, the maximum interincisal opening at the end of the follow-up period was equivalent to the preoperative measurements in both of the study groups, particularly in the hydrocortisone group.

The time course for trismus and associated restriction in oral function observed in the current study is consistent with results showing that trismus peaks on day 1 or day 2 postoperatively and often resolves by day 7 [22,27,28]. According to Cho et al., there was minimal trismus at the seven-day evaluation. Additionally, the highest subjective ratings for trismus, which were measured 48 hours after surgery, were improved by the seventh day, and these outcomes are consistent with the current study [29].

The use of corticosteroids as an irrigating solution appears to be a simple, painless, and cost-efficient technique that is helpful in minimizing postoperative sequelae. Overall, corticosteroids minimized inflammatory problems following third molar surgeries and had no major adverse reactions. These findings are consistent with the systematic reviews and meta-analyses published in the current literature [30,31].

Limitations

Due to the small sample size, it was difficult to generalize the results. Therefore, further studies are needed to evaluate the effect of hydrocortisone in managing postoperative pain, swelling, and trismus following the surgical removal of the third molar. Additionally, the efficacy of cold saline could have been evaluated in reducing postoperative pain, swelling, and trismus following the surgical extraction of the third molar.

Future recommendations

Hydrocortisone and povidone-iodine can be used routinely as an intraoperative irrigating solution during the surgical extraction of lower third molars, as they increase patient comfort, control swelling, and alleviate pain and trismus in the immediate postoperative period. The potential analgesic and anti-inflammatory properties of hydrocortisone make it a prospective contender for reducing the discomfort that patients usually experience in the immediate postoperative period.

## Conclusions

The present research has demonstrated that hydrocortisone, as an intraoperative irrigating agent, is beneficial in minimizing postoperative swelling within the first 48 hours. However, on the seventh day, there was no noticeable distinction between hydrocortisone, povidone-iodine, and normal saline. Hydrocortisone, on the other hand, performed effectively as an irrigating solution in controlling postoperative pain and trismus, followed by povidone-iodine and normal saline. This implies that using hydrocortisone as an irrigating solution is found to be a highly beneficial and inexpensive alternative in reducing the postoperative pain, swelling, and trismus caused by the surgical removal of impacted teeth.
